# Clinical and radiographic evaluation of triple antibiotic paste pulp therapy compared to Vitapex pulpectomy in non‐vital primary molars

**DOI:** 10.1002/cre2.434

**Published:** 2021-05-31

**Authors:** Ohoud T. Sijini, Heba J. Sabbagh, Khlood K. Baghlaf, Amina M. Bagher, Azzah A. El‐housseiny, Najlaa M. Alamoudi, Sara M. Bagher

**Affiliations:** ^1^ Department of Pediatric Dentistry King Abdulaziz University Hospital Jeddah Saudi Arabia; ^2^ Department of Pediatric Dentistry, Faculty of Dentistry King Abdulaziz University Jeddah Saudi Arabia; ^3^ Department of Pharmacology and Toxicology, Faculty of Pharmacy King Abdulaziz University Jeddah Saudi Arabia; ^4^ Department of Pediatric Dentistry King Abdulaziz University Jeddah Saudi Arabia

**Keywords:** deciduous teeth, endodontic treatment, primary molars and pulpectomy.

## Abstract

**Objectives:**

This study compared and evaluated the clinical and radiographic efficacy of non‐instrumentation triple antibiotic paste pulp therapy and Vitapex pulpectomy in non‐vital primary molars.

**Material and Methods:**

Healthy, 5–9 years old children with at least one non‐vital primary molar were included in the study. Molars were divided into two groups based on the subject's cooperation level. In the first group, molars received triple antibiotic paste, and a second group received Vitapex pulpectomy followed by a stainless‐steel crown. Triple antibiotic paste was freshly prepared and proportioned in equal parts by volume (metronidazole, minocycline, and ciprofloxacin = 1:1:1) before the scheduled treatment. A clinical and radiographic examination was performed by two trained and calibrated pediatric dentists at the pre‐operative baseline and the 6‐ and 12‐month follow‐up visits.

**Results:**

A total of 28 molars received triple antibiotic paste pulp therapy and 20 received Vitapex pulpectomy. At the 6‐month follow‐up, the success rate among the molars in the triple antibiotic paste group was clinically (92.85%) and radiographically (85.71%) higher compared to the Vitapex group (91.67%, 62.50% respectively) with *p* = 0.89 and 0.55 respectively. At the 12‐month follow‐up, the molars in the triple antibiotic paste group showed lower clinical (95.45%) but higher radiographic success rate (72.73%) compared to the Vitapex group (100% and 62.50%) with (*p* = 0.85 and 0.47) respectively. None of the differences were statistically significant.

**Conclusions:**

Both triple antibiotic paste and Vitapex can be clinically and radiographically effective in treating non‐vital primary molars.

## 1 INTRODUCTION

Primary teeth with infected root canals are a common problem, particularly in patients where the infection has reached the periradicular tissues (Takushige et al., 2004). Extraction and placement of a space maintainer is often suggested as a treatment option for primary teeth with infected root canals (Kayalvizhi et al., 2013). Primary teeth are considered a natural space maintainer and keeping them in the dental arch until exfoliation will safeguard children's proper dental, skeletal and psychological development (American Academy of Pediatric Dentistry Clinical Affairs Committee–Developing Dentition Subcommittee; American Academy of Pediatric Dentistry Council on Clinical Affairs, 2005; Raslan et al., 2017). Also, many undesirable consequences are associated with the early extraction of primary molars, including arch length loss, insufficient space for erupting premolars and mesial tipping of the permanent molars (Camp, 1994). Therefore, pulpectomy might be considered to preserve non‐vital primary teeth (American Academy of Pediatric Dentistry Clinical Affairs Committee–Developing Dentition Subcommittee; American Academy of Pediatric Dentistry Council on Clinical Affairs, 2005; Raslan et al., 2017).

A pulpectomy is a root canal procedure for irreversibly infected or non‐vital primary teeth (Pramila et al., 2016), in which the root canals are debrided, instrumented and then obturated with a resorbable material (Ahmed, 2014). Zinc oxide eugenol, iodoform‐based pastes, and a combination of iodoform‐based paste and calcium hydroxide (Vitapex) are common obturation materials (Kubota et al., 1992; Ozalp et al., 2005). Vitapex is a calcium hydroxide/iodoform paste and is delivered through a syringe with disposable tips (Ozalp et al., 2005). Although previous studies show favorable outcomes with Vitapex as a root canal filling material for primary teeth pulpectomy with a high clinical and radiographic success rate (Najjar et al., 2019; Nakornchai et al., 2010), the early and fast intra‐radicular resorption of the Vitapex creates a hollow tube in the root canals for bacteria to induce and cause re‐infection. Also, the Vitapex procedure is complex, long, requires an additional radiograph and the child's cooperation (Nakornchai et al., 2010; Nurko et al., 2000; Ozalp et al., 2005).

A newly developed concept of non‐instrumentation is lesion sterilization and tissue repair (LSTR) (Divya & Retnakumari, 2014; Takushige et al., 2004), involving a topical application of triple antibiotic paste (Reddy et al.) containing metronidazole, minocycline, and ciprofloxacin (Vijayaraghavan et al., 2012). TAP can sterilize non‐vital and infected pulp in both permanent and primary dentitions by being placed near the orifices after debriding the pulp chamber only and without preparing the radicular procedure (Asl Aminabadi et al., 2016; Hoshino et al., 1992). As a substitute for pulpectomy in the management of non‐vital primary molars, TAP promises a simpler, clinical, radiographic success rate (Pinky et al., 2011; Prabhakar et al., 2008).

This study aims to compare and evaluate the clinical and radiographic efficacy of non‐instrumentation triple antibiotic paste pulp therapy and Vitapex pulpectomy in non‐vital primary molars. Therefore, the null hypothesis is that there is no differences between clinical and radiographic efficacy of non‐instrumentation TAP pulp therapy and Vitapex pulpectomy in non‐vital primary molars. The study question is “is non‐instrumentation TAP pulp therapy an effective procedure for non‐vital primary molars.”

## 2 MATERIAL AND METHODS

### 2.1 Subjects

The sample size of 52 non‐vital primary molars was calculated using G‐Power 3.1.9.4 with a power of 80 and a risk/prevalence difference of 76% for TAP pulp therapy and 56% for Vitapex pulpectomy according to a 2010 study by Nakornchai et al. (2010).

Children receiving dental treatment at the pediatric dentistry clinics at King Abdulaziz University Faculty of Dentistry hospital (KAUFDH) between June 2017 and December 2018 were screened for eligibility. The screening took place during their initial examination or regular follow‐up appointments. The study was approved by the Research Ethics Committee at King Abdulaziz University (approval no. 067‐16) and registered on ClinicalTrial.gov with protocol ID# NCT04547764.

### 2.2 Subject inclusion criteria

The screening inclusion criteria included healthy cooperative children 5‐to‐9 years old with no allergies to any of the components of the dental materials used in the study and with at least one non‐vital primary molar that met the teeth inclusion criteria.

The children had a history of cooperative behavior during their previous dental treatment, which was classified according to Frankel's Behavioral Rating Scale and only those with cooperative or definitely cooperative behavior were included in the study (Stigers, 2016).

### 2.3 Teeth inclusion criteria

Primary molars with at least one of the following clinical and/or radiographic signs and symptoms were eligible and included in the study. Teeth inclusion criteria were decayed primary molar with clinical signs and symptoms of irreversible pulpitis necrosis and chronic infection such as spontaneous pain, fistula‐opening, tenderness to lateral and vertical percussion and palpation, and grade II or greater pathological tooth mobility. Radiographical signs included evidence of bifurcation radiolucency, periapical radiolucency, and pathological external or internal root resorption. Primary molars showing radiographic evidence of excessive internal or external root resorption, perforated pulpal floor, excessive bone loss in the furcation area involving the underlying tooth germ, and non‐restorable molars were excluded from the study. All included molars had physiological root resorption of less than one‐third of the root.

### 2.4 Clinical examination

Clinical examination and evaluation were performed by two trained and calibrated pediatric dentists independently of each other. Any disagreement between them was resolved by a third trained and calibrated examiner. If the examined primary molar met the clinical inclusion criteria, a standardized periapical radiograph was obtained for radiographic examination and evaluation. Clinical signs and symptoms recorded before the treatment were considered the pre‐operative baseline.

### 2.5 Radiographic examination

To facilitate the standardization and reproducibility of the X‐ray projection for the follow‐up visits radiographs, an XCP Rinn holder and an X‐ray beam centering system to allow accurate photostimulable phosphor (PSP) plates (Gendex Dental Systems, Hatfield, PA, USA) positioning and alignment were utilized. The X‐ray source Orix 70 (ARDET Dental & Medical Devices, Milano, Italy) was also standardized at 70 KV, 7 mA, exposure time 0.05 s. The PSP plates were then processed using the GXPS‐500 digital X‐ray phosphor plate system (GXPS‐500, Gendex Dental Systems, Hatfield, PA, USA). However, for the Vitapex group, we used bitewing radiographs that is placed apically to show the total root and allow measuring the root canal working length for master file selection. A digital film radiograph (MiPACS: Medicor Imaging, Charlotte, N.C., USA) with a digital holder to direct the X‐ray beam 90° between the primary molars using 65 KV, 7 mA, 0.064 s exposure time radiographic source.

The radiographs were scored on a 19‐in. screen by two trained and calibrated examiners O.S. and S.B. independently. In the event of disagreement, a third trained and calibrated examiner H.S. inspected the radiographs. The radiographic signs recorded before treatment were considered the pre‐operative baseline. An appointment was scheduled for all subjects who met the required inclusion criteria and were willing to commit for follow‐up visits. The nature and importance of the study were explained to the parents/legal guardian and an Arabic enrollment consent form was signed by the subject's parent/guardian before participation.

Before initiating the study, each of the examiners attended training and calibration sessions in which they were asked to perform a radiographic evaluation of 10 randomly selected non‐vital primary molars independently, twice, 2 weeks apart, to calculate the radiographic inter and intra‐reliability among the examiners. For the clinical examination, only the interexaminer's reliability was calculated by examining 10 randomly selected non‐vital primary molars by the examiners independently.

### 2.6 Clinical procedure

Eligible primary molars were allocated to receive either TAP pulp therapy (study group) or Vitapex pulpectomy (controls group) based on the cooperation level of the subject after a detailed explanation of both procedures. Patients with definite positive behavior were enrolled in the Vitapex group because they could better tolerate the longer and more complex procedure.

### 2.7 Preparation of TAP

The antibiotics used in the preparation of TAP were metronidazole tablets (500 mg) (Flagyl™, Sanofi‐Aventis), minocycline tablets (105 mg) (Vulga™, Jazeera Pharmaceutical Industries), and ciprofloxacin tablets (250 mg) (Ciprobay™ 250, Bayer). The enteric coating of the tablets was removed using a sterilized sharp blade, and the tablets were pulverized with a sterilized mortar and pestle. The powdered antibiotics were transferred into three sterile amber‐colored airtight glass containers and stored in refrigerators. The TAP was freshly prepared by a pharmacist before each scheduled treatment appointment. For its preparation, powdered antibiotics were proportioned in equal parts by volume (metronidazole, minocycline, and ciprofloxacin = 1:1:1), and then mixed with normal saline to get a paste‐like consistency. Unused TAP was discarded.

### 2.8 Clinical steps

**FIGURE 1:**
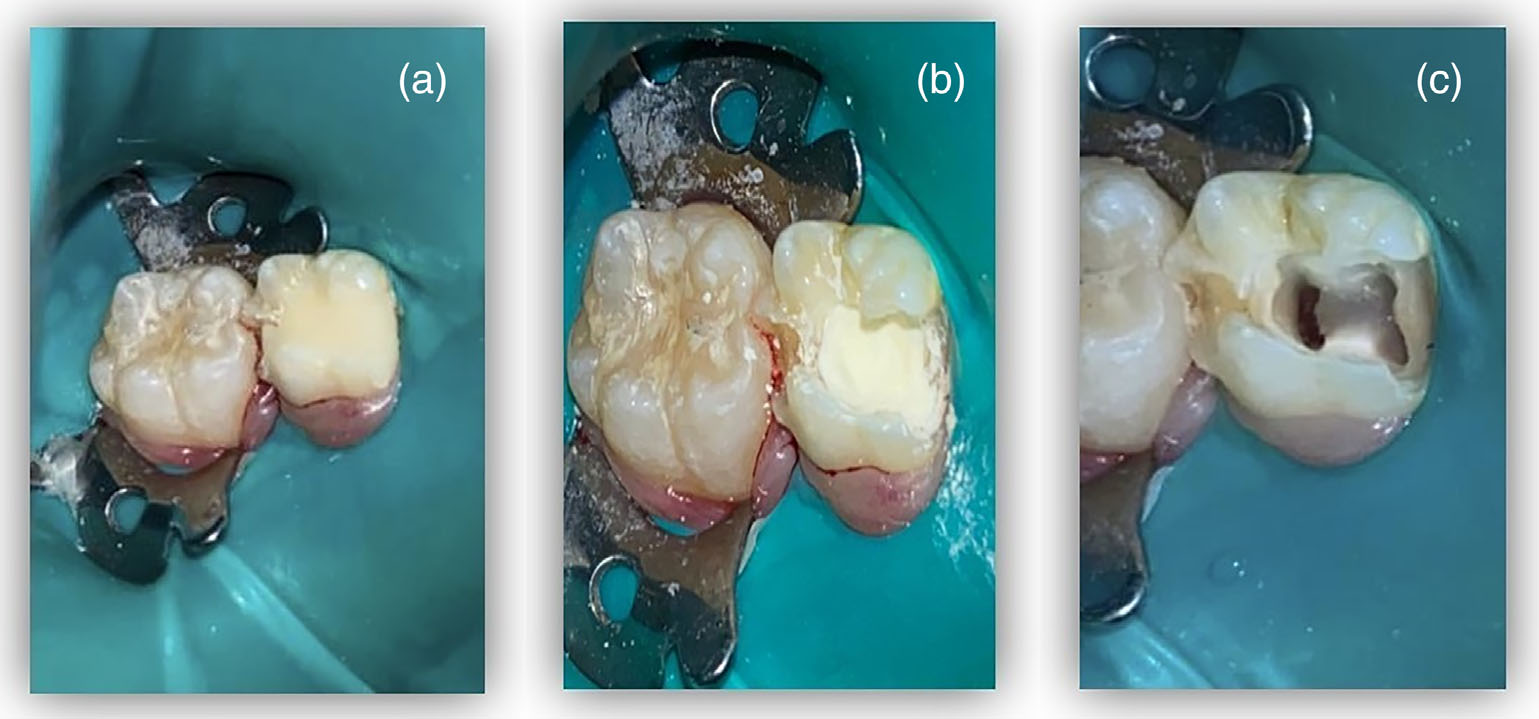
Tooth treated with TAP pulp therapy showing the procedure of treatment; (a) tooth after access opening and coronal pulp amputation, (b) lesion sterilization and application of freshly mixed TAP and (c) glass ionomer restoration applied and covered with stainless steel crown

All clinical procedures were performed by pediatric dentist postgraduate students at KAUFDH under the supervision of three calibrated pediatric dentist consultants (OS, HS, SB). Included primary molars were anesthetized and isolated with a rubber dam. After complete caries removal and access cavity preparation by a sterile fissure bur in a high‐speed handpiece, the non‐vital coronal pulp tissue was removed using a sterile sharp spoon excavator or a sterile low‐speed round bur. The orifices of the root canals were enlarged 1–2 mm by a sterile low‐speed small‐size round bur and irrigated with 2.5% sodium hypochlorite (NaOCl). If hemorrhage persisted, sterile cotton pellets of 5% NaOCl were applied against the pulp floor for 1 min. The cavity was then dried with cotton pellets and the TAP placed on the enlarged root canal orifices and pulpal floor. Light‐cured glass‐ionomer cement (Vitrebond; 3 M ESPE) was placed and the primary molars were reinforced immediately by a stainless‐steel crown (SSC) (3M; ESPE Stainless Steel Primary Molars Crowns) (see Figure 1).

For the Vitapex group, primary molars were anesthetized and isolated with a rubber dam during the first visit. After complete caries removal, the access cavity was prepared in the same way as in the TAP group's primary molars, but the non‐vital pulp was removed completely from the coronal and radicular part of the tooth. Root working length was determined to be 2 mm shorter than the radiograph apex measured in the pre‐operative radiograph. Cleaning and shaping the root canal were carried out using K‐files (Dentsply Maillefer, Ballaigues, Switzerland) in a pullback direction to a maximum size of 30–40 with continuous irrigation of 2.5% NaOCl. Sterile paper points were used to dry the canals. Vitapex (DiaDent Group International, Burnaby, B.C., Canada) was injected into the canal by a pre‐packed syringe. A sterile cotton pellet was placed in the pulp chamber and sealed with Cavit (ESPE America., Norristown, PA, USA) as a temporary filling. Two weeks later, the root canals were irrigated with 2.5% NaOCl and dried using sterile paper points. Vitapex was injected into the canal and then light‐cured glass‐ionomer cement (Vitrebond; 3M ESPE) was placed and the primary molars were reinforced by SSC.

### 2.9 Clinical and radiographic follow‐up visits

All clinical and radiographic examinations and evaluations were performed by two trained and calibrated examiners and any disagreement between them was resolved by a third trained and calibrated examiner. During the clinical examination and evaluation, the examiners were blind to which group the molar belonged. Blindness was not achievable radiographically.

At the follow‐up visits, treated primary molars that had healed completely from any reported clinical signs and symptoms at the pre‐operative baseline, such as spontaneous pain, fistula‐opening, tenderness to lateral and vertical percussion and palpation, and grade II or greater pathological tooth mobility, were considered a success. Radiographically, any treated primary molar with diminished or completely healed bifurcation/periapical radiolucency, no progression of pathological external/internal root resorption and no newly developed radiographic lesion were considered successful.

The clinical evaluations were compared to the pre‐operative baseline and were carried out at 3‐, 6‐, and 12‐month follow‐up visits. Radiographic evaluation was performed at 6‐ and 12‐month follow‐up visits following the American Academy of Pediatric Dentistry recommendation for dental radiographic prescription in children (American Academy of Pediatric Dentistry, 2017). However, the clinical and radiographic signs and symptoms were documented only for the research at the 6‐ and 12‐month follow‐up visits. If one or more of the previously mentioned clinical and radiographic signs and symptoms of failure were present at any of the follow‐up visits, the primary molar was extracted and excluded from the recall visits. However, they were not excluded from the analysis and were considered as failure in the next follow‐up visit. Subjects that failed to come in for follow‐up visits were excluded from the paired nominal data analysis.

### 2.10 Statistical analysis

Data were entered and analyzed using SPSS Statistical analyses version 22.0 (SPSS, Chicago, II, USA). Descriptive analysis was reported in frequencies and percentages. The success rate comparison of both groups at 6‐ and 12‐month follow‐ups was determined by statistical analysis with Fisher's exact test. McNemar's test was used for paired nominal data. A *p*‐value of 0.05 or less was considered statistically significant.

## 3 RESULTS

The agreement of the single examiner at pre‐operative baseline and 12‐month follow‐up was strong (baseline κ = 0.95 and final follow‐up κ =0.91). The inter‐examiner reliability between the examiners (pre‐operative baseline κ =0.90 and final follow‐up κ =0.75) also indicated substantial to strong agreement.

Seventy eligible subjects with at least one non‐vital primary molar and who met the subject's inclusion criteria were evaluated during the duration of the study. Fourteen primary molars were excluded either because they did not meet the tooth clinical and radiographic inclusion criteria or their families declined to participate in the study.

**FIGURE 2:**
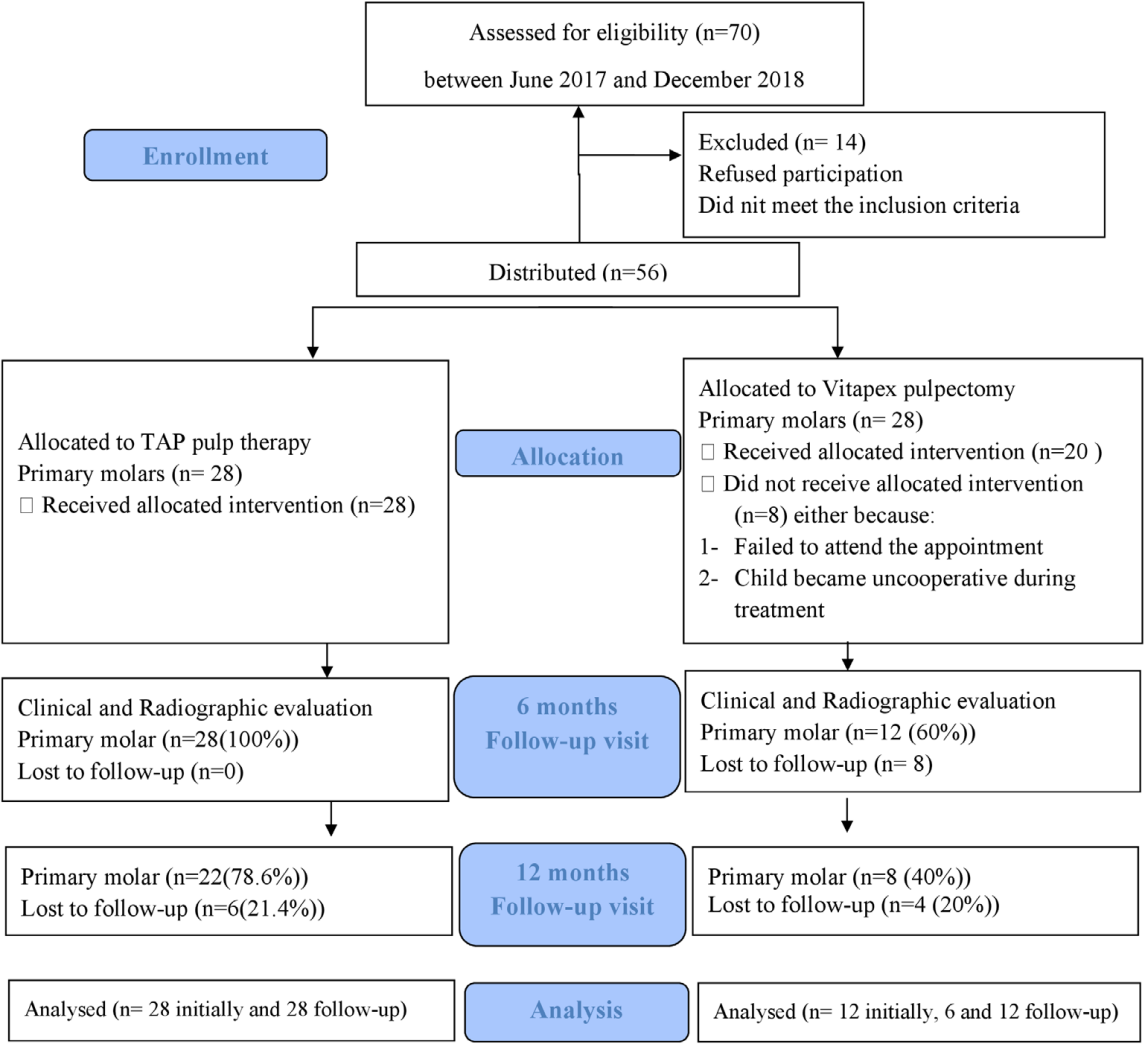
Study flow chart

The remaining 56 eligible non‐vital primary molars in 53 subjects were divided into either the TAP pulp therapy group or the Vitapex pulpectomy group, based on the child's level of cooperation. In the Vitapex group, eight molars were excluded because the patient either failed to attend the scheduled treatment appointment or the child became uncooperative during the treatment and refused to complete it. Finally, a total of 48 non‐vital primary molars (28 in the TAP pulp therapy group and 20 in the Vitapex pulpectomy group) in 45 subjects (21 [46%] males, 24 [54%] females) continued the study. Figure 2 presents the study flow chart. There was no statistically significant difference in the gender distribution between both groups at the pre‐operative baseline with 16 (57.14%) males in the TAP group and 13 (65%) females in the Vitapex group (*p* = 0.15).

For the type of primary molars, 26 (92.85%) of the TAP group and 17 (85%) of the Vitapex pulpectomy group were second primary molars. Based on the arch location, most of the included primary molars in both groups were in the lower arch. The distribution of the primary molars in both groups is presented in Table 1.

**TABLE 1:** Distribution of primary molars included in the study (*n* = 48)

Molar type	TAP (*n* = 28)	Vitapex group (*n* = 20)	*p*‐value*
	*N* (%)	N (%)	
First primary molar	2 (7.14)	3 (15)	0.73
Second primary molar	26 (92.85)	17 (85)	
**Arch location**			
Upper primary molar	8 (28.57)	6 (30)	1
Lower primary molar	20 (71.42)	14 (70)	

* *p* value < 0.05 significant‐using fisher exact test.

At the pre‐operative baseline examination and evaluation, none of the included primary molars in both groups showed radiographic evidence of pathological internal or external root resorption. Bifurcation radiolucency was seen in 15 (53.57%) and 11 (55%) of the TAP pulp therapy group and Vitapex pulpectomy group, respectively. The distribution of the pre‐operative baseline clinical signs and symptoms and the radiographic signs between both groups are presented in Table 2. There was no statistically significant difference reported between both groups in respect to the pre‐operative baseline clinical signs and symptoms and radiographic signs except for the presence of fistula‐opening, with statistically significantly more primary molars with fistula‐opening on the TAP pulpotomy group 14 (50%) compared to the Vitapex pulpectomy group 4 (20%) (*p* = 0.05) (see Table S1).

**TABLE 2:** Clinical and radiographic evaluation at the baseline, 6‐ and 12‐months follow‐up visits in both groups

Signs and symptoms	TAP group *N* (%)					Vitapex group *N* (%)				
	Baseline (*n* = 28)	6 months (*n* = 28)	*p*‐value	12 months (*n* = 22)	*p*‐value	Baseline (*n* = 12)	6 months (*n* = 12)	*p*‐value	12 months (*n* = 8)	*p*‐value
**Clinical sign and symptoms**										
Spontaneous pain	13 (46.42)	1 (3.57)	0.001*	1 (4.54)	0.001*	5 (45)	0	0.05*	0	<0.001*
Fistula‐opening	14 (50)	0	< 0.001*	0	<0.001*	4 (20)	0	0.27	0	0.29
Pain to percussion (lateral)	12 (42.8)	1 (3.57)	0.003*	0	0.001*	0	1 (8.33)	0.37	0	0.281
Pain to percussion (vertical)	12 (42.85)	0	0.001*	0	0.001*	2 (16)	0	0.24	0	0.029*
Pathological mobility	3 (10.71)	0	0.24	0	0.24	0	0	1	0	1
**Radiographic signs**										
Bifurcation radiolucency	15 (53.57)	3 (10.71)	0.0021*	3 (13.63)	0.013*	4 (33)	2 (16.66)	0.47	3 (37.5)	0.068
Periapical radiolucency	5 (17.86)	1 (3.57)	0.134	2 (9.09)	0.44	1 (10)	1 (8.33)	0.678	1 (12.5)	1
External resorption	0	1 (3.57)	1	2 (9.09)	1	0	1 (8.33)	0.375	1 (12.5)	0.31
Internal resorption	0	1 (3.57)	1	2 (9.09)	1	0	1 (8.33)	0.375	1 (12.5)	0.31

* Statistically significant *p*‐value <0.05, *p* value calculated according to McNemar test .

Clinically, a statistically significant decrease in the number of treated primary molars with spontaneous pain, and pain associated with vertical percussion among the TAP pulp therapy and Vitapex pulpectomy groups at the 6‐ and 12‐month follow‐up visits (*p* < 0.001) compared to the pre‐operative baseline was recorded. Radiographically, a significant decrease in bifunctional radiolucency in the TAP pulp therapy group at the 6‐ and 12‐month follow‐up visits compared to the pre‐operative baseline was detected (*p* < 0.001).

At the 6‐month follow‐up visit, a total of 40 (83.33%) out of 48 molars in 37 subjects were available for clinical and radiographic evaluation with a dropout of 8 (16.67%) compared to the pre‐operative baseline. Twenty‐eight (100%) out of 28 in the TAP pulp therapy group and 12 (60%) out of 20 in the Vitapex pulpectomy group presented for the 6‐month follow‐up visit. The remaining eight cases in the Vitapex group that failed to come in were those who reported spontaneous pain and bifurcation involvement. At 6 months, one primary molar in both TAP pulp therapy (3.57%) and Vitapex pulpectomy (8.33%) groups failed both clinically and radiographically. Also, among the TAP pulp therapy group, four (14.29%) primary molars failed radiographically and two (7.14%) failed clinically. In the Vitapex pulpectomy group, only one (8.33%) primary molar failed clinically and two (16.67%) failed radiographically. Primary molars with any clinical and/or radiographic failure were not excluded from the 12‐month follow‐up visit analysis but were quoted as a failure.

**FIGURE 3:**
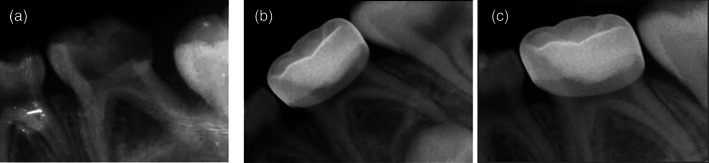
Successful TAP pulp therapy in a lower second molar with deep carious lesion and bifunctional radiolucency. (a) Pre‐operative baseline radiograph. (b) Six‐months follow‐up radiograph showing reduction in the size of the bifurcation radiolucency. (c) Twelve‐months follow‐up showing complete healing of the radiographic radiolucency

**FIGURE 4:**
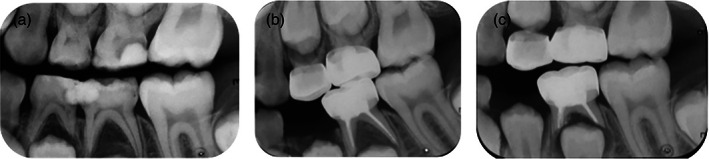
Successful Vitapex pulpectomy treatment in a lower second molar with deep carious lesion. (a) Pre‐operative baseline radiograph. (b) Six‐months follow‐up radiograph showing reduction in the size of the bifurcation radiolucency. (c) Twelve‐months follow‐up

At the 12‐month follow‐up visit, a total of 30 (62.5%) out of 48 molars in 27 subjects were available for clinical and radiographic examination with a drop out of 18 (37.5%) compared to the pre‐operative baseline. Twenty‐two (78.57%) out of 28 in the TAP pulp therapy group and eight (40%) out of 20 in the Vitapex pulpectomy group presented for the 12‐month follow‐up visit. One (4.55%) primary molar in the TAP pulp therapy group presented with only clinical signs and symptoms of failure and six (27.27%) failed radiographically only. In the Vitapex pulpectomy group, none of the presented primary molars showed signs or symptoms of clinical failure but three (37.5%) failed radiographically. Thus, a total number of six out of 22 primary molars (27.27%) in the TAP pulp therapy group and three out of eight (37.5%) in the Vitapex pulpectomy group showed clinical and/or radiographic signs and symptoms of failure. No statistically significant differences in the clinical and radiographic success rate between the two groups at the 6‐ and 12‐month follow‐up visits were detected (Table 3). Figures 3 and 4 represents radiographic pictures of a successfully treated primary molar.

**TABLE 3:** Clinical and radiographic success rates of TAP and Vitapex treatment at 6‐ and 12‐months follow‐up visits

Evaluation criteria	Follow‐up visits	*N* (%)		*p*‐value
		TAP*	Vitapex**	
**Clinical sign and symptoms**	**6 months**	26 (92.85%)	11(91.67%)	**0.89**
	**12 months**	21 (95.45%)	8 (100%)	**0.55**
**Radiographic signs**	**6 months**	24 (85.71%)	10 (83.33%)	**0.85**
	**12 months**	16 (72.73%)	5 (62.50%)	**0.47**

* TAP pulp therapy group: At 6 months follow‐up visit *N* = 28, at 12 months follow‐up visit *N* = 22.

** Vitapex pulpectomy group: At 6 months follow‐up visit *N* = 12, at 12 months follow‐up visit *N* = 8.

## 4 DISCUSSION

Our study investigated the success rate of the simple non‐instrumentation TAP pulp therapy conducted on children with some behavioral challenges and found it to be similar to the complex two‐visit Vitapex pulpectomy, among non‐vital primary molars.

Pulp therapy on pediatric patients is usually associated with numerous challenges, including behavior management, various morphology of primary teeth and the complexity of root canal treatment (Ahmed, 2013). A Cochrane Database Systematic Review assessed the effects of different pulp treatment techniques and associated medicaments for the treatment of extensive decay in primary teeth (Nadin et al., 2003). They found many included trials had no clinical or radiological failures in either trial arms, and the overall proportion of failures was low. The evidence suggests that MTA may be the most efficacious medicament to heal the root pulp after pulp treatment of a deciduous tooth; however, the cost of MTA may preclude its clinical use (Nadin et al., 2003).

Vitapex pulpectomy is a lengthy and complicated procedure usually requiring two treatment visits and is not always suitable for children. On the other hand, the use of TAP pulp therapy requires removing only the coronal pulp tissue, followed by permanent restoration, which is usually performed in a single visit and requires no mechanical instrumentation. Therefore, TAP pulp therapy was introduced as an alternative procedure to pulpectomy, especially for very young or uncooperative children and in areas with limited resources (Zacharczuk et al., 2019).

Due to the previously mentioned advantages of TAP pulp therapy, the Vitapex pulpectomy in our study was performed mainly on very cooperative children while less cooperative children received TAP pulp therapy. Still, there were difficulties with a few children who became uncooperative and the Vitapex pulpectomy could not be completed. Difficulty in recalling or completing the treatment of pulpectomy in children was also reported in previous studies (Dean, 2015; Najjar et al., 2019).

One of the reported advantages of TAP pulp therapy is that it can be effective and successful for primary molars with poor prognosis and with advanced root resorption on which conventional pulpectomy is not indicated (Raslan et al., 2017; Takushige et al., 2004). In our study, only primary molars with less than one third root resorption were included in the study. Also, one of the main problems associated with Vitapex pulpectomies in primary molars is the material's fast resorption (Nakornchai et al., 2010; Nurko et al., 2000; Ozalp et al., 2005). The material's rate of resorption in the present study and its influence on the clinical and radiographic success rate was not evaluated. Also, we have to consider that Vitapex is an industrial ready‐to‐use syringe application system, which facilitates the manipulation of the material. The TAP pulp therapy used in this study was freshly prepared at the time of use, which raised questions about the duration of the therapeutic activity of the prepared TAP, its ability to penetrate the infected radicular pulp tissue and its effect on the overall clinical and radiographic success rate of the treatment (Raslan et al., 2017; Takushige et al., 2004).

Triple antibiotic paste pulp therapy has been used in several studies in the pulpectomy treatment of primary molars (Nakornchai et al., 2010; Pinky et al., 2011; Raslan et al., 2017; Takushige et al., 2004). The paste consists of three antibacterial agents – metronidazole, minocycline, and ciprofloxacin – that have different acting mechanisms and different spectra of activity. Metronidazole is a broad‐spectrum antibiotic agent against anaerobic bacteria, which binds to DNA and disrupts its helical structure. Minocycline is a semi‐synthetic tetracycline that inhibits protein synthesis. It also exhibits a broad spectrum of activity against gram‐negative and gram‐negative bacteria. Ciprofloxacin is a fluoroquinolone that inhibits the enzyme DNA gyrase of bacteria. It, too, exhibits potent bactericidal activity against gram‐negative bacteria, but it has limited efficacy against gram‐positive bacteria. A combination of these drugs results in the successful eradication of root canal diverse bacteria flora and prevents the development of antibiotic resistance. Radiographically, these antibiotics showed bone healing (Nakornchai et al., 2010; Pinky et al., 2011; Raslan et al., 2017; Takushige et al., 2004), but the reason behind the regeneration needs to be investigated in future histological studies. We suggest that bone regeneration could be due to the antibacterial effect of the antibiotics that eradicated bone inflammation or it might have a direct bone regeneration effect.

Previous clinical studies compared the success rate of different TAP combinations in the pulpectomy of primary molars (Pinky et al., 2011; Raslan et al., 2017; Takushige et al., 2004). In a 2017 study by Raslan et al. (2017), minocycline was replaced by clindamycin. In other studies, metronidazole was substituted with either ornidazole (Doneria et al., 2017; Nanda et al., 2014; Pinky et al., 2011), or with tinidazole (Jaya et al., 2012), and all these TAP combinations reported favorable clinical and radiographic success.

At the pre‐operative baseline, all signs and symptoms were distributed similarly in TAP pulp therapy and Vitapex pulpectomy groups, with no statistically significant differences except for the frequency of fistula‐opening, which was significantly reported more often among the TAP pulp therapy group. However, this could add to the success rate of TAP pulp therapy.

Although in our study TAP pulp therapy was applied to the pulp orifices with no intracanal instrumentation, other studies have included instrumentation and removal of the radicular pulp tissue (Prabhakar et al., 2008; Reddy et al., 2017). These studies reported a very high clinical and radiographic success rate at 12 months following treatment. This might be attributed to the instrumentation and complete extirpation of both non‐vital coronal and radicular pulp tissue.

The current study reported a clinical success rate for Vitapex of 91.67% and 100% at 6 and 12 months, respectively, and radiographic success of 83.33% and 62.50% at 6 and 12 months, respectively. Our findings are consistent with previous studies reporting a clinical success rate of the Vitapex pulpectomy on primary molars ranging between 80.4% to 100% (Nakornchai et al., 2010; Nurko et al., 2000; Ozalp et al., 2005; Pramila et al., 2016), and a radiographic success rate ranging between 56% to 100% at 12 months after treatment (Chen et al., 2017; Nakornchai et al., 2010; Nurko et al., 2000; Ozalp et al., 2005; Trairatvorakul & Chunlasikaiwan, 2008).

In agreement with Nakornchai et al. (2010), we found no significant difference in both the clinical and radiographic success rates between the TAP pulp therapy and the Vitapex pulpectomy at the 6‐ and 12‐month follow‐up visits. Twelve months after treatment, the clinical success rate in our study was found to be 95.45%, which is higher than the percentages reported by previous studies (Nakornchai et al., 2010; Prabhakar et al., 2008). Those studies reported a 93% to 100% success rate at the 12‐month follow‐up visits. However, the clinical success rate reported in our study was higher than the clinical success rate reported by a recent study at the 18‐month follow‐up visit (87.5%) (Zacharczuk et al., 2019).

The radiographic success rate of TAP 12 months after treatment was 72.73% in our study, which is slightly less than the radiographic success reported by previous studies (Nakornchai et al., 2010; Zacharczuk et al., 2019). They reported a radiographic success rate higher than 75% at the 12‐month (Nakornchai et al., 2010), and 18‐month follow‐up visits (Trairatvorakul & Chunlasikaiwan, 2008; Zacharczuk et al., 2019). This could be due to differences in the radiographic evaluation criteria, as previous studies considered a statistic radicular or bifurcation radiolucency a success. However, in our study, only radiographic lesions that decreased in size or were completely healed at the follow‐up visits compared to the pre‐operative baseline were considered successful (Nakornchai et al., 2010).

Radiographically, the most common cause of failure was internal root resorption and an increase in the bifurcation radiolucency (Doneria et al., 2017; Pinky et al., 2011; Prabhakar et al., 2008). Our results show that a total of 6 (27.27%) out of the 22 primary molars that received TAP pulp therapy failed after 12 months due to either internal root resorption and/or an increase in the bifunctional and periapical radiolucency.

In 2005, a large‐scale retrospective study by Moskovitz et al. (2005) reported a statistically significant higher failure rate with the second primary molars compared to the first primary molars pulpectomies. This failure rate was also reported to be associated more with mandibular than with maxillary primary molars (Moskovitz et al., 2005). On the other hand, a recent retrospective study evaluated radiographic and clinical prognosis of pulpectomy on different primary molars and reported an insignificant slight increase in the frequency of failures associated with first molars compared to the second (Mendoza‐Mendoza et al., 2017). Due to the limited number of first primary molars included in the study, we could not evaluate the difference in the success rate between the first and second primary molars.

Consolidated Standards of Reporting Trials (CONSORT) guidelines were followed by this study except for four of its items (8 to 11) that discussed allocation and randomization. The main limitations of our study are that 15 subjects in the Vitapex group were not allocated to intervention either because they did not attend the scheduled treatment appointment, preferred extraction, or became uncooperative during the treatment and had to be excluded from the study. The problem of selecting an appropriate sample in the children population is a daily challenge, so a larger research population was difficult to achieve. Additionally, as with any follow‐up study, the attrition of participants was relatively high. Although this might result in bias in the estimation effect of the material, in our study the main attrition occurred in the control group rather than the TAP group. Therefore, the TAP success rate should be unaffected. Also, all of the cases that failed to come from the Vitapex group are those reporting severe clinical and radiographic signs and symptoms as spontaneous pain and bifurcation involvement. Thus, this limitation might favor the outcome of Vitapex over TAP.

Furthermore, it was not possible to distribute participants randomly into the two analyzed groups. This is because Vitapex required higher cooperation from both the child and parents, having been conducted over two visits and is more complicated compared with TAP. However, these criteria for patient distribution might also result in a bias favoring the outcome of Vitapex over TAP. As for the study concealment, it is not possible to blind the examiner for radiographic examination since the complete pulpectomy obturation of Vitapex will look different than the TAP pulp therapy that is only in the pulp chamber.

Further randomized clinical trial studies with a larger sample size and long term clinical and radiographic evaluation until the exfoliation of the treated primary molars is recommended to evaluate the influence of TAP pulp therapy of the treated primary molar exfoliation and the underlying permanent succedaneous teeth. Also, it is recommended to evaluate the influence of the stage of physiological root resorption on the clinical and radiographic success rate of TAP pulp therapy.

## 5 CONCLUSION

From the results of this study and within the limitations of the study and follow‐up durations, the following conclusions were obtained:

- Both Vitapex pulpectomy and non‐instrumentation triple antibiotic paste pulp therapy can be effective clinically and radiographically in the management of non‐vital primary molars to prolong their survival until natural exfoliation.
- No significant difference between the success rate of the non‐instrumentation TAP pulp therapy and the Vitapex pulpectomy in non‐vital primary molars 6 and 12 months after treatment.

## CONFLICT OF INTEREST

The authors declare no potential conflict of interest.

## AUTHOR CONTRIBUTIONS

All authors contributed either in writing the paper, supervising and carrying out the treatment, preparing the three mix antibiotics, recruiting the data, examining and analysing the data. All Authors had read, approved and agreed to the submission of the manuscript.

## Supporting information


**Table S1.** Clinical sign and symptoms and radiographic signs of TAP pulp therapy and Vitapex pulpectomy at pre‐operative baseline, 6‐ and 12‐months follow‐up visits.

## Data Availability

The data that support the findings of this study are available on request from the corresponding author. The data are not publicly available due to privacy or ethical restrictions.
