# Supplementary material for: Clinical and radiographic evaluation of triple antibiotic paste pulp therapy compared to Vitapex pulpectomy in non‐vital primary molars
Source: Clin Exp Dent Res. 2021 May 31;7(5):819–28. doi: 10.1002/cre2.434 (PMC8543458; doi:10.1002/cre2.434)
Supplement: Supplementary file 1 — Table S1. Clinical sign and symptoms and radiographic signs of TAP pulp therapy and Vitapex pulpectomy at pre‐operative baseline, 6‐ and 12‐months follow‐up visits. [file CRE2-7-819-s001.docx]

**Supplementary table 1.** Clinical sign and symptoms and radiographic signs of TAP pulp therapy and Vitapex pulpectomy at pre-operative baseline, six- and 12-months follow-up visits.

| **Sign and Symptoms** | **Pre-operative Baseline (n=48)** | | | | | | **6 months follow-up (n=40)** | | | | | | **12 months follow-up (n=30)** | | | | |
| --- | --- | --- | --- | --- | --- | --- | --- | --- | --- | --- | --- | --- | --- | --- | --- | --- | --- |
|  | **TAP**  **(n=28)**  **N(%)** | **Vitapex**  **(n=20)**  **N(%)** | | ***P*-value** | | | **TAP**  **(n=28)**  **N(%)** | **Vitapex (n=12)**  **N(%)** | | ***P*-value** | | | **TAP (n=22)**  **N(%)** | | **Vitapex (n=8)**  **N(%)** | | ***P*-value** |
| **Clinical sign and symptoms** | | | | | | | | | | | | | | | | | |
| Spontaneous pain | 13(46.42) | | 12(60) | | 0.39 | 1(3.57) | | | 0(0.00) | | 1 | 1(4.54) | | 0 | | 1 | |
| Fistula | 14(50) | | 4(20) | | 0.041* | 0(0.00) | | | 0(0.00) | | 1 | 0 | | 0 | | 1 | |
| Pain to percussion (lateral) | 12(42.85) | | 5(25) | | 0.24 | 1(3.57) | | | 1(8.33) | |  | 0 | | 0 | | 1 | |
| Pain to percussion (vertical) | 12(42.85) | | 9(45) | | 1 | 0(0.00) | | | 0(0.00) | | 1 | 0 | | 0 | | 1 | |
| Abnormal mobility | 3(10.7) | | 0(0.00) | | 0.255 | 0(0.00) | | | 0(0.00) | | 1 | 0 | | 0 | | 1 | |
| **Radiographic signs** | | | | | | | | | | | | | | | | | |
| Bifurcation radiolucency | 15(53.66) | | 11(55) | | 1 | 3(10.71) | | | 2(16.66) | | 0.63 | 3(13.63) | | 3(37.5) | | 0.3 | |
| Periapical radiolucency | 5(17.86) | | 2(10) | | 1 | 1(3.57) | | | 1(8.33) | | 1 | 2(9.09) | | 1(12.5) | | 1 | |
| External resorption | 0(0.00) | | 0(0.00) | | 1 | 1(3.57) | | | 1(8.33) | | 1 | 2(9.09) | | 1(12.5) | | 1 | |
| Internal resorption | 0(0.00) | | 0(0.00) | | 1 | 1 (3.57) | | | 1(8.33) | | 1 | 2(9.09) | | 1(12.5) | | 1 | |

*Statistically Significant *P*-value < 0.05 calculated according to fissure exact test.
